# Sepsis: Inflammation Is a Necessary Evil

**DOI:** 10.3389/fcell.2019.00108

**Published:** 2019-06-20

**Authors:** Christina Nedeva, Joseph Menassa, Hamsa Puthalakath

**Affiliations:** Department of Biochemistry and Genetics, La Trobe Institute for Molecular Science, La Trobe University, Melbourne, VIC, Australia

**Keywords:** inflammation, sepsis, apoptosis, programmed cell death, immune suppression

## Abstract

Sepsis is one of the leading causes of deaths world-wide and yet there are no therapies available other than ICU treatment. The patient outcome is determined by a complex interplay between the pro and anti-inflammatory responses of the body i.e., a homeostatic balance between these two competing events to be achieved for the patient’s recovery. The initial attempts on drug development mainly focused on controlling inflammation, however, without any tangible outcome. This was despite most deaths occurring during the immune paralysis stage of this biphasic disease. Recently, the focus has been shifting to understand immune paralysis (caused by apoptosis and by anti-inflammatory cytokines) to develop therapeutic drugs. In this review we put forth an argument for a proper understanding of the molecular basis of inflammation as well as apoptosis for developing an effective therapy.

## Introduction

Early medical records have documented infectious diseases in humans as far back as 1000 BC, and yet, pathogenic infection remains as the leading cause of morbidity and mortality (Ruffer and Ferguson, 1911; Cossart, 2014). Infection leading to sepsis continues to be one of the biggest health problems world-wide. Although difficult to discern the absolute global burden of the disease, it is estimated that thirty million people are affected each year (Reinhart et al., 2013). The disease predominantly affects low- to middle-income countries and is responsible for an estimated six million deaths (Fleischmann et al., 2016). In addition, every year one million deaths of newborns are due to maternal/neonatal sepsis (Vogel, 2017). In the United States alone, costs associated with this disease can exceed $16 billion dollars, as most patients admitted to ICU require mechanical ventilation to stay alive (Angus et al., 2001).

Despite the heavy cost of sepsis, the etiology of the disease continues to be enigmatic. In the past, it was believed that the primary source of infection originated solely from the gut microbiota (Friedman et al., 1998). However, subsequent studies showed that *Pseudomonas* sp. that colonizes and causes infection of the upper respiratory tracts was the most commonly associated infection in sepsis (Rangel-Frausto, 1999; Mayr et al., 2014). Now we know that sepsis is a highly heterogenous disease both in terms of its cause and its progression. Before the 90s, the majority of septic patients who presented at the clinic showed gram-negative organisms in their blood (Polat et al., 2017). This lead some scientists to establish diagnostic criteria for the sepsis syndrome – claiming specific medical symptoms and known cause of infection are central for diagnosis (Bone et al., 1989). Within the following decade it became evident that although gram-negative bacteria are still prevalent in septic patients, gram-positive microbiota became more apparent within patient sera (Friedman et al., 1998). In fact, almost the same number of gram-negative and gram-positive bacteria are today associated with the disease (Vincent and Abraham, 2006). However, the causative agent is not always bacteria as parasites and fungus can also cause sepsis (Hubner et al., 2013; Florescu and Kalil, 2014; Liang, 2016). Furthermore, in about a third of patients an infectious pathogen is not detectable (Bone et al., 1989; Liang, 2016). This includes trauma patients whom frequently displayed clinical signs of sepsis but lacked bacteria in the blood (Goris et al., 1985). These discrepancies forced physicians to modify the diagnostic criteria for sepsis in 1992 at a Consensus Conference in Chicago (Bone et al., 1992). These new criteria suggested that infection did not have to be limited to bacteria and systemic inflammatory response syndrome – SIRS – became the new age term to describe the disease (Bone et al., 1992).

Although diagnostic criteria were being updated regularly – one aspect of sepsis drew the attention of researchers and remained constant - the presence of inflammation during disease. The inflammatory nature of sepsis was investigated as far back as 1960 – where the first clinical trial commenced to attenuate the inflammatory response (Bennett et al., 1962). These studies led to the use of corticosteroids; however, no therapeutic benefit was noted (Bennett et al., 1962). Drug trials which target the inflammatory phase of sepsis would continue well into the 2000s without any tangible benefits in patient survival (Polat et al., 2017). A recent shift in the paradigm would lead researchers to believe that inflammation is in fact necessary to fight infection associated with disease (Ding et al., 2018). Nevertheless, these revelations are relatively new and therapies to treat the disease are still under investigation.

## Role of Inflammation in Sepsis Pathology: a Double-Edged Sword

**FIGURE 1:**
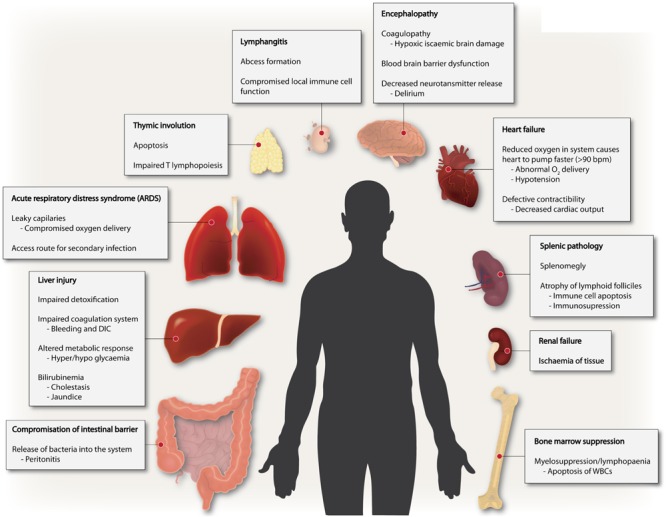
Sepsis is a multi-faceted disease. Multiple derangements exist in sepsis involving several different organs ranging from altered coagulation, immune suppression to inflammation and multiple organ failure.

Sepsis is fundamentally an inflammatory disease mediated by the host immune response. The innate immune response is facilitated by the activation of pattern recognition receptors (PRR) during early sepsis. The receptor-response is highly dynamic and can be elicited by both pathogen-associated molecular patterns (PAMPs) and/or damage-associated molecular patterns (DAMPs) such as mitochondria released from injured tissues (Mogensen, 2009; Hauser and Otterbein, 2018). At an organism level, complement, surface-receptors of epithelial, endothelial and disseminated immune surveillance cells incite such responses (Takeuchi and Akira, 2010). Intracellular signaling process is highly complex – with complementary and/or redundant roles for numerous signaling pathways, ultimately leading to expression of genes involved in adaptive immunity and inflammation. However, the deregulated hyperinflammation can lead to the many symptoms seen in the early phase of sepsis including disseminated intravascular coagulation (DIC) and subsequent multi-organ dysfunction syndrome (MODS), inflammation-coagulation due to aberrant platelet activation, peripheral vasodilation leading to low blood pressure ensuing hypoperfusion of the kidney and kidney failure (Dhooria et al., 2016; Wang et al., 2018). Thus, sepsis is a multifaceted disease manifested in many ways including endocrine disorder, coagulopathy, polyneuropathy, complement activation and polyneuropathy, all emanating from dysregulated inflammation (Figure 1).

Inflammation is an essential step in alerting the immune system to the presence of infection so that the hosts white blood cells can quickly locate and combat the pathogen (Weighardt et al., 2000). This response is typically tightly controlled, with inflammation waning after infection is resolved – returning to basal levels with the host’s white blood cells following suit. When homeostasis is maintained, excessive inflammation and immune cell activity is avoided, and the immune system can prime itself for effective response to future infection (Newton and Dixit, 2012). During sepsis, the stimulus that is recognized by the immune system, ranging from PAMP’s like endotoxins and viruses to DAMP’s during serious trauma, is far greater than in regular infections (Hotchkiss et al., 2016). The immediate result is a cytokine storm brought on due to the overstimulation of the numerous white blood cells that recognize those factors. This dysregulation in response causes a myriad of symptoms that make sepsis distinctly different to regular infections, regardless of severity (Martin, 2012). When functioning normally, the immune system can combat most infections, with an imperceptible amount of inflammation occurring before the pathogens are cleared from the host. Resolving most infections so rapidly, with little damage to the host, depends on the strict regulation of cytokines. Cytokines are essential in the process of initiating and escalating the innate immune response as well as the adaptive immune response (Banyer et al., 2000). However, high levels of inflammatory cytokines can co-exist with a significant innate immune suppression, which can lead to nosocomial infections (Hall et al., 2013).

## Molecular Mechanisms of Inflammation

Many different antigenic constituents from bacteria, viruses and fungi, as well as tissue trauma are known causative agents of sepsis. Common pathogens recurrently isolated from septic patients include gram-positive *Staphylococcus aureus* and *Streptococcus pneumoniae* and gram-negative bacteria *Escherichia coli* and *Pseudomonas aeruginosa* (Martin et al., 2003). PAMPs such as LPS are recognized by toll-like receptors (TLRs) expressed on antigen presenting cells (APCs), such as macrophages and dendritic cells (Poltorak et al., 1998). APCs express a variety of these TLRs containing leucine-rich repeats, which act to sense and elicit responses against these antigens (Kawai and Akira, 2010). Upon receptor contact with their cognate ligands, pro-inflammatory intermediates are recruited, some of which include mitogen activated protein kinases (MAPKs) – which are activated upon phosphorylation – signal transducers and activators of transcription (STAT), Janus kinases (JAK) and nuclear factor κ (kappa)-light-chain-enhancer of activated B cells (NF-κB) – which translocates to the nucleus. As a result, gene expression is initiated to promote inflammatory cytokine and chemokine production (Johnston et al., 1995). This fine-tuned process is dependent on the repertoire of PAMPs, DAMPs and signaling pathways stimulated, to determine intensity and route of response, in an effort to re-establish host homeostasis. In the septic response, excessive inflammation due to deregulated intrinsic mechanisms is associated with pathology (Surbatovic et al., 2013).

## Immune Activation Genes

The transcription complex, NF-κB, is triggered in response to numerous extracellular inflammatory stimuli (Sen and Baltimore, 1986; Pahl, 1999). Activation of NF-κB by post-translational mechanisms induces expression of early activation genes including IL-1/12/18 and type-1 IFNs – to name a few (Naumann and Scheidereit, 1994). These inflammatory cytokines initiate synthesis of other cytokines and chemokines, such as IL-6/8, IFN-γ and CXC-chemokine ligands – exacerbating the inflammatory response. Stimulation of PRRs leading to the inflammatory cascade causes adaptive immune constituents to either become reactive or suppressive (Hayden et al., 2006). Such canonical pathways have shown to instigate the hyperinflammation observed in sepsis. Hence, studies have aimed to block NF-κB – as well as other intermediates – to attenuate hyper-responsiveness, however, results are conflictive (Sha et al., 1995; Gjertsson et al., 2001). Studies investigating differentially expressed genes in sepsis demonstrate genetic aberrations associated with disease, which could potentially be used as diagnostic markers (Prabhakar et al., 2005; Zhang et al., 2017). Interleukin-1 receptor-associated kinase 3 (IRAK3) is one such marker, which is specifically elevated in blood monocytes of septic patients and can possibly possess diagnostic value (Escoll et al., 2003).

## Endocrinopathy

Sepsis is a highly inflammatory disorder with the presence of organ dysfunction in severe cases and mostly caused by bacterial infection (Bone et al., 1989). These obvious characteristics of the disease prompted galvanize the belief that inflammation solely was responsible for sepsis related mortality. This claim was supported by endotoxemia models, which were deemed appropriate as they recapitulate obvious pathogenic features of the disease (Heppner and Weiss, 1965; Hardaway et al., 1996; Deitch, 2005). Hence, therapies were designed to attenuate host inflammatory responses evident in sepsis. One of the first anti-inflammatory treatments was the use of corticosteroids (Bennett et al., 1962). Evidence of adrenal gland insufficiency in patients with sepsis initially encouraged the use of steroids (Melby and Spink, 1958). Indeed, endotoxemia animal models of disease supported these findings (Mckechnie et al., 1985) and led to the use of steroids in a human study. The trial consisted of septic patients administered with high doses of methylprednisolone, leading to significant reduction in mortality with (Schumer, 1976). In a subsequent study, high dose steroid administration was found to have adverse effects (Warrington and Bostwick, 2006). Also, with adequate vasopressor therapy and fluid resuscitation (Colling et al., 2018), the use of steroids for treating sepsis became obsolete. Recently, a meta-analysis re-visited the applicability of steroids in sepsis – suggesting that low doses could be advantageous (Rochwerg et al., 2018). Another larger randomized study – comprised of 3800 subjects – measured survival in septic shock patients infused with hydrocortisone. They concluded that hydrocortisone did not reduce 90-day mortality when compared to placebo (Venkatesh et al., 2018). As it stands, the benefits of steroid use for treating sepsis remain vague and lack promise.

## Coagulation Cascade

Coagulopathy associated with sepsis has long been identified as a clinical feature of disease (Martinez et al., 1966). Of those who present to the clinic, 35% meet criteria for DIC, which is a robust predictor of mortality (Wheeler and Bernard, 1999; Bakhtiari et al., 2004). During early DIC, activation of thrombin leads to the formation of fibrin complexes followed by thrombocytopenia. Late progressive DIC is characterized by the deposition of fibrin in the small blood vessels of the body, causing dissemination of micro-thrombi, which is associated with organ failure (Taylor et al., 2001; Gando et al., 2016). In order to prevent mortality in septic patients with microthrombus development, studies have used high-dose anti-thrombin therapy, however, no benefit was noted with the treatment (Warren et al., 2001). Other studies investigating the antithrombotic activity of heparin – unfractionated or low-dose – have also showed lack of effectiveness in preventing sepsis-related mortality (Zhang and Ma, 2006; Jaimes et al., 2009). The biggest drawback of such studies seems to be their single facet approach, where in fact a multi-facet approach is required for treating heterogeneous disease such as sepsis. Also to be taken into consideration is the close relationship between the innate immune system and the coagulation cascade (Esmon, 2003). Ample evidence suggest that hemostatic changes in sepsis can be regulated by pro-inflammatory mediators such as TNF-α during the “cytokine storm” (Zimmerman et al., 2002; Levi and Van Der Poll, 2010). Hence, the pathological “cross-talk” between coagulation and inflammation during septic shock warranted further investigation. In this context, the coagulation mediator, activated protein C, readily became of interest as a treatment option as this protein has important roles in coagulation and in attenuating immune responses (Opal, 2004). Identification of this protein as a putative therapeutic target for sepsis led to the controversial Recombinant Human Activated Protein C Worldwide Evaluation in Severe Sepsis (PROWESS) study (Bernard et al., 2001). Recombinant human activated protein C, marketed by Eli Lilly as Drotrecogin alpha activated (DrotAA) or Xigris, was used in this study. However, the study showed a modest 6.1% decrease in 28-day mortality in severe septic shock patients treated with DrotAA (Bernard et al., 2001). Similar studies using DrotAA in Early Stage Severe Sepsis (ADDRESS) and Extended Evaluation of Recombinant Human Activated Protein C United States Trial (ENHANCE), showed lack of drug efficacy with increased side effects such as hemorrhage (Abraham et al., 2005; Vincent et al., 2005).

Translation from “bench to bedside” appears to be the biggest hurdle for researchers in the development of successful treatments for sepsis. The disconnect has indeed been highlighted in the failure of past drug trials, especially those impeding inflammatory pathways associated with disease. However, in recent years researchers have shifted their focus to immune activation in sepsis – as inflammation is critical for clearing infection. Hence, immune stimulating strategies reveal an innovative focal point for treating sepsis pathogenesis.

## Cytokine and Complement Activation

Cytokines TNF-α and IL-1 are the most extensively studied pro-inflammatory mediators in sepsis. These cytokines are capable of activating target immune cells to produce additional inflammatory mediators and as a consequence, a heightening immune responses. This prompted an increased focus on these cytokines to develop a therapeutic strategy to treat sepsis (Schulte et al., 2013). Other cytokines with anti-inflammatory property such as IL-6, IL-8, IL-12, IFN-γ, and IL-10 could dampen the inflammatory response (Van Der Poll and Opal, 2008). The cytokine predominantly produced by Th17 T-cells, IL-17, possesses the capacity to provoke a pro-inflammatory immune response by eliciting the production of TNF-α, which in turn provides a route for cross-talk between lymphocytes and phagocytes (Weaver et al., 2007). Murine studies have shown that blockage of IL-17 is associated with marginal survival advantage (Flierl et al., 2008). The sepsis inflammatory response has also been shown to be regulated by macrophage migration inhibitory factor (MIF). MIF has been shown to be vital for the regulation of host immune responses via modulation of TLR4. Mice lacking MIF have been shown to have a defective response to intravenous LPS introduction, due to reduced TLR4 expression (Calandra et al., 2000).

Apart from the production of inflammatory cytokines, complement activation is also associated with sepsis onset. This in turn has profound effects on coagulation, compromising the endothelial barrier integrity transitioning into a pro-coagulant state. Similar to cytokines, complement activation is also initiated by PAMPs and DAMPs. During sepsis, complement peptide C5a is converted to a potent chemo-attractant state, causing derangement in neutrophil function, that results in tissue damage (Guo and Ward, 2005). Additionally, this potent peptide amplifies inflammatory responses by stimulating production of pro-inflammatory cytokines, which is thought to contribute to organ failure in acute sepsis (Ward, 2010). Furthermore, complement factors have also been detected in clinical settings of disease suggesting a role in sepsis pathogenesis. However, treatment methods to prevent complement activation are ineffectual in reducing mortality associated with sepsis (Markiewski et al., 2008).

## Immune Cell Death

**FIGURE 2:**
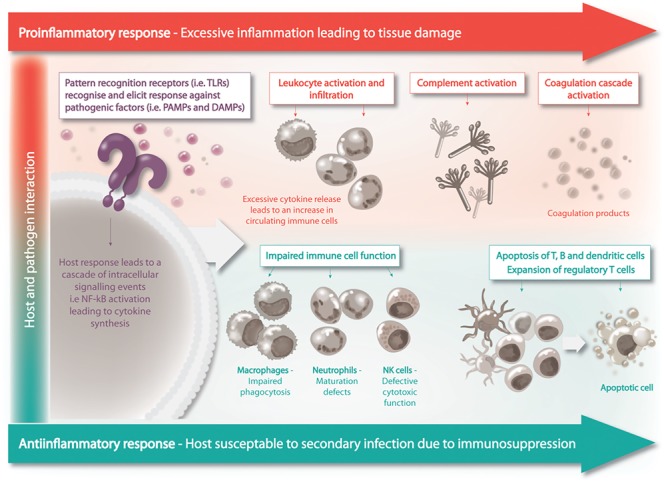
Sepsis is a biphasic disease, the initial phase is characterized by overwhelming inflammation followed by immuno-suppression. A homeostatic balance between these two competing events is to be achieved for the patient’s recovery.

Sepsis pathogenesis was believed to consist of two distinct phases in response to systemic infection, which included the initial pronounced inflammation phase – or cytokine storm – that transitioned into a phase of prolonged immune suppression (Hotchkiss et al., 2013). Patients who survive the initial phase can enter a protracted hypo-inflammatory phase know as PICS – persistent inflammation/immunosuppression and catabolism syndrome (Figure 2). PICS is characterized by organ failure, persistent inflammation, protein catabolism/cachexia, ineffectual wound healing and increased susceptibility to infection due to immune suppression (Gentile et al., 2012). The propensity for patients with sepsis to readily develop persistent, recurrent and nosocomial infections suggested the existence of immune suppression in sepsis (Grimaldi et al., 2011). In further support of this idea, patients with sepsis have a higher rate of latent virus reactivation and blood cultures positive for opportunistic organisms (Otto et al., 2011; Walton et al., 2014). Irrespective of the disease classification, the immune suppression and dysregulation associated with disease is undeniably the major cause of sepsis related fatalities (Daviaud et al., 2015).

During sepsis, cells from both the innate and adaptive immune system are affected. The immune cells displaying marked depletion during sepsis include B cells, CD4^+^, and CD8^+^ T cells and dendritic cells in lymphoid organs such as the thymus, spleen and lymph nodes (Hotchkiss et al., 2005). Apoptosis, both extrinsic and intrinsic, has proved to be the driver of this depletion, with dying cells positive for active caspases and enhanced expression of pro-apoptotic BH3-only proteins. Multiple studies have shown, using various models such as transgenic mice and caspase-inhibitors, that blockade of lymphocyte apoptosis improves survival in sepsis (Hotchkiss et al., 1999a; Peck-Palmer et al., 2009). Indeed, autopsy studies of patients with sepsis revealed that immune cell apoptosis was the underlying cause of mortality (Torgersen et al., 2009). Furthermore, survival had a strong negative correlation with immune cell apoptosis during sepsis in mice (Hotchkiss et al., 1999b). The apparent linear relationship between disease severity and apoptosis is due to less circulating immune cells, hence, decreased surveillance and detection of infectious pathogens. In turn, compromising the hosts ability to successfully clear what should be a “mild” secondary infection. The negative effects of cell death during sepsis also impacts apoptotic cell uptake and clearance by surviving immune cells. Loss of follicular dendritic cells causes considerable impairment of T and B cell function, with CD4^+^ T cell deficit impeding macrophage activation (Tinsley et al., 2003). Consequently, impaired macrophages are unable to mount the suitable inflammatory response toward the invading agent.

## Immune Cell Tolerance and Dysfunction

Lymphocytes undergoing apoptosis – during sepsis – can also serve to further suppress immune functions via interactions with macrophages, monocytes or dendritic cells. Phagocytotic cells are triggered to release anti-inflammatory cytokines – such as IL-10 and TGFβ – upon engulfment of apoptotic cells rendering them anergic. Additionally, this process can cause aberrations at the transcriptional level – preventing pro-inflammatory cytokine production – thus further contributing to immune paralysis (Coopersmith et al., 2003). Immune tolerance caused by excessive exposure to endotoxin can similarly have major consequences on macrophage functionality. In addition to excess release of immunosuppressive mediators, endotoxin tolerant macrophages possess relatively low levels of HLA-DR on their surface, resulting in a lack of antigen presentation (Saenz et al., 2001). Malfunction of sentinel first line defense immune cells combined with pronounced apoptosis is associated with a poor outcome in sepsis (Huang et al., 2009). Innate defenses are further compromised during disease pathogenesis due to impaired function of neutrophils and natural killer (NK) cells. During sepsis, circulating neutrophils exhibit an immature phenotype affecting transmigration, adhesion and the formation of neutrophil extracellular traps (NETs) (Kovach and Standiford, 2012). Indeed, neutrophils isolated from patients with sepsis showed to lack maturity, evidently having chemotaxis defects and reduced oxidative capacity. The depressed effector functions alter neutrophil antimicrobial defenses and is reported to be associated with the development of secondary infections in *in vivo* and in clinical settings (Demaret et al., 2015). This is also the case with NK cells, which are heavily depleted during sepsis. Animal and human studies have shown that as well as being reduced in number, remaining NK cells have defective cytotoxic function (Forel et al., 2012). Amongst the vast magnitude of cellular aberrations occurring during sepsis, exhaustion of T cells is characteristic of prolonged septic insult. Onset of T cell exhaustion is caused by a high load of antigen and amplified levels of anti- and pro-inflammatory cytokines, characteristic of the host septic environment. A recent study using cecal-ligation and puncture (CLP) showed that exhaustion of CD8^+^ T cells can extend beyond initial septic insult and can inflict long-lasting changes in T cells leading to compromised reactivity toward future infections (Condotta et al., 2015). Hence, detrimental effects of immune cell death and dysregulation result in a long-term immunological “scar” causing substantial mortality of patients many years later when initial disease has been resolved.

## Drug Trials for Sepsis: a Catalog of Failures

Severe immune dysregulation is the prominent hallmark of sepsis – rendering the disease biologically complex and consequently a challenge to treat (Sprung et al., 2006). More than 100 clinical trials have investigated putative treatments for sepsis, yet a cure still remains elusive (Table 1). Past failures of clinical trials can largely be attributed to disinclination of researchers to abandon ineffectual sepsis models. For instance, multiple past trials were based on studies which used experimental mice dosed with abnormal amounts of pathogen to mimic sepsis (Fink, 2014). Models such as this have since been largely discredited as they cause inflammation at a supra-physiological level (Lewis et al., 2016). The use of such imprecise models to study sepsis – in-turn – led to the development of non-targeted drugs which were unable to resolve disease in the clinic.

**Table 1:** Sepsis therapy, a catalog of failures.

Target	Strategy	References
Lps/Endotoxin	HA-1A	Ziegler et al., 1991
	E5531	Bunnell et al., 2000
	Anti-CD14	Reinhart et al., 2004
	Eritoran	Opal et al., 2013
	Polymyxin B	Payen et al., 2015
	conjugate	
Endocrinopathy	Methylprednisolone	Bone et al., 1989
	Vasopressin	Ohsugi et al., 2016
Hypercoagulability /Disseminated Intravascular Coagulation (DIC)	Activated Protein C	Bernard et al., 2001
	Anti-thrombin	Warren et al., 2001
	Heparin	Zhang and Ma, 2006
	Thrombomodulin	Hagiwara et al., 2016

Cytokines	Anti-TNF-α	Tracey et al., 1987
	IL-1 receptor	Fisher et al., 1994
	Antagonists	
	Soluble TNF-α receptor	Borrelli et al., 1996
	Diacerhein	Calisto et al., 2012
Eicosanoids	Ibuprofen	Bernard et al., 1997
Nitric Oxide	L-NMMA	Petros et al., 1994
Oxidat1ve Stress	Statins	Patel et al., 2012
	Selenium	Sakr et al., 2014
Nf-Kb Transcription	Curcumin	Zhong et al., 2016
Apoptosis	Caspase inhibitors	Weber et al., 2009

*Most, if not all, were targeting inflammation including caspase inhibitors. Caspases do have a central role in inflammation (Mandal et al., 2018).*

In the late 1960s, many researchers began to trial immunomodulatory agents for the treatment of sepsis (Davis et al., 1969). One of the most potent immune activators that gained prominence was bacterial cell wall component – LPS (Okeke and Uzonna, 2016). Studies demonstrated that lethality, associated with high doses of endotoxin in mice, was reverted with IgG infusion (Davis et al., 1969; Rubenstein and Worcester, 1969). Subsequently, the first anti-endotoxin trial commenced, which investigated the level of antiserum in patients infected with gram-negative bacteria. The study showed a reduction in mortality upon bacterial vaccination (Ziegler et al., 1982). Promising pre-clinical results led to the development of the monoclonal antibody – HA-1A – directed against the toxic lipid A component of LPS. Initial human trials showed that patients with sepsis tolerated the antibody well and were thought to benefit from treatment (Ziegler et al., 1991). However, lack of data reproducibility raised doubts against the study leading to a second trial. Further investigation revealed that the HA-1A treated groups had an increase in mortality – leading to drug withdrawal from the market (Costongs et al., 1993; Mccloskey et al., 1994). Homologs of LPS have also been designed to antagonize the activity of endotoxin at the receptor level. One such study used potent LPS antagonist E5531, which blocked endotoxin response in healthy volunteers infused with small amounts of LPS (Bunnell et al., 2000). However, LPS homologs lost credibility when applied in a clinically relevant setting of sepsis. A study using eritoran – a synthetic TLR4 antagonist – demonstrated the drug’s inability to reduce 28-day mortality in septic patients (Opal et al., 2013). The lack of effectiveness seen with anti-endotoxin treatment is perhaps not surprising since only about half of septic patients present with gram-negative infections. This suggested that pre-clinical models of disease do not appropriately reflect the heterogeneity of the human condition (Hotchkiss and Karl, 2003; Buras et al., 2005). Additionally, studies have used adoptive transfer of bone marrow cells between LPS-sensitive and LPS-resistant mice, which found that transferred bone marrow cells rendered mice susceptible to endotoxin lethality (Michalek et al., 1980). However, this study added credence to the fact that endotoxin wasn’t killing the mice directly, rather their response to that exposure was.

For the last two decades, anti-cytokine strategies were thought to have boundless therapeutic potential. However, despite this optimism, they’ve shown to have little use in the treatment of sepsis. This was the case for extensively studied adjunctive therapies targeting tumor necrosis factor or TNF-α. Neutralization of this target receptors entailed the use of monoclonal antibodies as well as soluble TNF-α receptors as decoy receptors (Tracey et al., 1987; Vacheron et al., 1992; Borrelli et al., 1996). Many of these studies showed promise in rodents, however, could not demonstrate the same effect in human clinical trials. A notable study performed by Fisher et al., 1996, revealed that targeting inflammatory mediators could even be harmful. In this randomized, double-blinded study, septic patients were administered with recombinant soluble TNF-α receptor. Recombinant protein did not reduce mortality in septic patients and high doses were even associated with increased mortality (Fisher et al., 1996. In fact, clinical use of anti-TNF-α therapy has been linked to increased risk of infections (Ali et al., 2013). On the contrary, studies have shed light on the benefit of cytokine activation during sepsis (Echtenacher et al., 2001). Other studies have similarly used IL-1 receptor antagonists to test prognostic value in clinical sepsis, yet, fail to demonstrate significant reduction in mortality (Fisher et al., 1994). Failed trials that target inflammation in sepsis highlight the disconnect between laboratory experiments and clinical outcome and this warrants an urgent recalibration in research approach.

## Current Trials

Many recent therapeutics have targeted endotoxins and cytokines circulating through patients with sepsis at dangerously high levels, in an attempt to reduce the inflammation and the associated pathology (Polat et al., 2017). However, all these therapies, while showed promise in animal models, showed no change in patient outcome or overall survival, and in some cases increased mortality. These therapeutics often had the desired effect of reducing the levels of its target endotoxin or cytokine, however, they consistently failed to improve short term clinical outcomes observed during SIRS i.e., organ failure, elevated heart rates and blood pressure or longer-term outcomes such as: time spent in ICU, rate of opportunistic infection and mortality. The failure to improve patient outcome by any of these measures’ highlights both the complexity of the disorder as well as the flaw of targeting the initial acute phase of sepsis. Lack of correlation between cytokine levels and mortality was highlighted in a retrospective study published 20 years ago (Antonelli, 1999). Recently, focus has shifted to the chronic immunosuppressive phase as the cause of most sepsis-related deaths.

The subsequent immunosuppressive phase of sepsis is characterized by a drop in pro-inflammatory cytokine levels and leukopenia, frequently culminating in infection by opportunistic pathogens and death (Polat et al., 2017). During sepsis both myeloid cells and lymphocytes undergo high levels of apoptosis, with many of the remaining cells entering a state of anergy, rendering both the innate and adaptive arms of the immune system ineffective (Hotchkiss et al., 2013). The magnitude of the drop of total and functioning leukocytes is directly correlated with patient survival. Coupled with consistently falling mortality rates during the acute phase of sepsis (Levy et al., 2010), increasing the number of healthy leukocytes presents a promising therapeutic target that is now beginning to be explored (Meisel et al., 2009; Chousterman and Arnaud, 2018). Immuno-stimulatory adjuvant therapies intend to counter the immune-paralysis that occurs in the chronic phase of sepsis (Meisel et al., 2009; Chousterman and Arnaud, 2018; Francois et al., 2018). These therapies aim to reduce apoptosis of leukocytes allowing their numbers to increase and revert them to a functional phenotype. Such therapies currently being investigated include the growth factors granulocyte-macrophage colony stimulating factor (GM-CSF) and IL-7 and the receptor, programmed cell death 1 (PD-1).

GM-CSF is a potent cytokine that stimulates the generation and maturation of monocytes and neutrophils, allowing them to effectively respond to pathogens (Mathias et al., 2015; Shindo et al., 2015). This effect has been demonstrated both *ex vivo* and *in vitro*. Addition of recombinant human GM-CSF to whole blood of septic patients recapitulates their phenotype closer to that of a healthy person. Treatment re-sensitizes both neutrophils and macrophages to LPS, with treated cells releasing significantly higher levels of pro inflammatory cytokines including TNF-α, IL-6, and IL-8, all of which are released at significantly lower levels in many patients during the later stages of sepsis (Mathias et al., 2015). Another marker of immune cell dysfunction is prolonged downregulation of membrane-associated human leukocyte antigen receptors (mHLA-DR) (Landelle et al., 2010). Lower expression levels of mHLA-DR have been associated with poorer outcomes and lower patient survival (Landelle et al., 2010), with GM-CSF demonstrating the capacity to restore mHLA-DR expression. A clinical trial involving patients with severe sepsis or septic shock conducted by Meisel et al. (2009) corroborated these findings. Patients were initially treated with 4 μg/kg daily for the first five days, then depending on the response, given 4 or 8 μg/kg daily for the next 3 days. *Ex vivo* analysis of monocytic function demonstrated that the monocytes from patients treated with GM-CSF – that were then stimulated with LPS – secreted higher levels of TNF-α, IL-6, and IL-8. Additionally, mHLA-DR expression was significantly upregulated, compared to that of normal levels, and less of the anti-inflammatory cytokine IL-10 was expressed (Meisel et al., 2009). Analysis of patient serum revealed that absolute neutrophil and monocyte count increased by a factor of four, with all patients approaching a normal white blood cell count after treatment. Additionally, TNF-α levels were increased, however, all other cytokines remained unchanged relative to the placebo group. Other clinical outcomes included treated patients spending less time on mechanical ventilation and reduced APACHE-II scores. Despite these favorable short- and long-term changes to many clinical outcomes, 28-day mortality was not reduced (Meisel et al., 2009).

IL-7 is a growth factor that stimulates the proliferation and maturation of many cell types, in particular T lymphocytes. IL-7 also causes many desirable changes in T lymphocytes (that may prove beneficial) in the context of sepsis disease progression, including: upregulation of Bcl-2 proteins and resistance to apoptosis, proliferation and enhanced function (Francois et al., 2018). During sepsis both CD4^+^ and CD8^+^ T cell populations drop considerably, and, like myeloid cells, the magnitude of the drop is correlated closely with patient survival (Drewry et al., 2014). In a phase II trial investigating CYT107, a recombinant form of human IL-7 in treating patients with septic shock and severe lymphopaenia (Francois et al., 2018). The most significant finding of this study was that, despite the complexity of the inflammation and immunosuppression seen during sepsis, there was a four-fold increase in absolute T lymphocyte count, which persisted well beyond the completion of therapy (Francois et al., 2018). However, much like the trial of GM-CSF, there was no significant difference in 28- or 90-day survival.

One of the main contributing factors of lethality in sepsis is immune tolerance, the mechanisms of which are only beginning to be understood. One pathway in which this occurs is the upregulation of PD-1 on T lymphocytes and PD-L1 specifically on APC’s (Watanabe et al., 2018). During the immunosuppressive phase of sepsis, APCs upregulate PD-L1 further impairing remaining T-lymphocytes and compounding the effects of the suppressive cytokine profile of patients (Shindo et al., 2015; Liu et al., 2017). When T cells expressing PD-1 interact with cells expressing high levels of PD-L1, any response the T cells would have otherwise mounted is suppressed. This is compounded by the elevated levels of soluble PD-L1 in the serum of septic patients, leading to further lymphocyte attrition (Liu et al., 2017). PD-1 and PD-L1 antagonists are a new class of therapeutic blocking the interaction between the two molecules (Patera et al., 2016). These monoclonal antibodies are being investigated for use in diseases such as cancer and types of chronic viral infection, where restoring T cell function is of particular importance in fighting the disease (Watanabe et al., 2018). Similar to GM-CSF and IL-7 treatment, the PD-L1 antagonist BMS-936559 is well tolerated, with all therapies having little to no adverse effects when used to treat critically ill patients (Patera et al., 2016). Importantly, all drugs did not elicit an excessive pro inflammatory cytokine response, that would have further harmed patients (Meisel et al., 2009; Francois et al., 2018; Watanabe et al., 2018).

However, these therapies are not without limitations. The absence of improved short-term survival in all immunoadjuvant therapies is the most glaring shortcoming. It is likely due to a complex variety of reasons, though they do offer some clear benefit to sepsis patients. These limitations include the relatively small sample size in all the studies, the severity of sepsis of those included, as well as the use of 28- or 90-day survival as the end point. Severe sepsis and septic shock have the highest mortality rates of all types of sepsis during the acute phase, chronic phase and long after discharge from hospital (Karlsson et al., 2009). Long term mortality in these cases is over 1.5 times higher than in-hospital mortality, with the quality of life of survivors also being lower (Karlsson et al., 2009). It is these deaths that immuno-stimulant adjuvant therapies may offer the greatest benefits i.e., in reducing immune scarring and allowing the survivors of sepsis to reconstitute a functioning immune system. Past trials have not been powered to follow patients for such extended periods of time, but it is possible that is where the greatest benefits will be seen.

## Concluding Remarks

Experimental drug therapies for sepsis are at cross-roads with the withdrawal of the latest drug Xigris (activated protein C, Eli Lilly) from the market following the negative results of the 1,700-person PROWESSSHOCK phase III trial in 2011. Critical-care physicians now have no drugs specifically approved to treat severe sepsis with the failure of *Talactoferrin alfa* (an immunomodulatory lactoferrin, Agennix, Germany) and AstraZeneca’s *CytoFab*, an antibody directed against pro-inflammatory tumor necrosis factor-alpha (TNF-α) to name a few. Apart from using incorrect animal models (such as endotoxin-mediated sepsis in the absence of any confirmed infection), these failures could be attributed to the strategy of targeting inflammation, notwithstanding the fact that inflammation contributes to less than 20% of sepsis-related mortality. In the context of sepsis, inflammation is necessary evil as inflammatory cytokines are the activators of both the innate and the adaptive immune systems. Blocking of this pathway proven to be counterproductive in treating sepsis as there is a clear correlation between anti-inflammatory therapies and increased risk of infections (Ali et al., 2013). Use of steroids is yet another controversial topic. Since its inception in 1976 (Schumer, 1976), glucocorticoids are the preferred choice of treatment by great many physicians in spite of the fact that it does not offer any survival advantage (Venkatesh et al., 2018). There is a collective imperative on both the researchers and the physicians to measure the treatment outcome in terms of patient survival rather than reduced economic cost in ICUs. The immune paralysis phase of sepsis accounts for more than 80% of the sepsis-related mortalities and there is an inverse correlation between immune cell apoptosis and patient survival (Hotchkiss et al., 2013). Methods for identifying when patients have entered the immunosuppressive phase of sepsis and for detecting defects in immunity might enable the application of potent new immunotherapies. Therefore, there is a need to identify the factors that lead to immune-suppression by deregulated cytokine production and immune cell apoptosis. To understand the molecular mechanism of immune cell death during sepsis, particular emphasis should be given on the intrinsic or the Bcl-2 family mediated apoptosis.

## Author Contributions

All authors listed have made a substantial, direct and intellectual contribution to the work, and approved it for publication.

## Conflict of Interest Statement

The authors declare that the research was conducted in the absence of any commercial or financial relationships that could be construed as a potential conflict of interest.
